# Improvement of sciatic nerve regeneration by multichannel nanofibrous membrane-embedded electro-conductive conduits functionalized with laminin

**DOI:** 10.1007/s10856-022-06669-0

**Published:** 2022-05-31

**Authors:** Niloofar Nazeri, Mohammad Ali Derakhshan, Korosh Mansoori, Hossein Ghanbari

**Affiliations:** 1 Cellular and Molecular Research Center, Qazvin University of Medical Sciences, Qazvin, Iran; 2 Department of Medical Nanotechnology, School of Advanced Technologies in Medicine, Tehran University of Medical Sciences, Tehran, Iran; 3 Department of Medical Nanotechnology, School of Advanced Medical Sciences and Technologies, Shiraz University of Medical Sciences, Shiraz, Iran; 4 Neuromusculoskeletal Research Center, Iran University of Medical Sciences, Tehran, Iran; 5 Research Center for Advanced Technologies in Cardiovascular Medicine, Cardiovascular Diseases Research Institute, Tehran University of Medical Sciences, Tehran, Iran

**Keywords:** Electrospinning, PLGA, CNT, Nanofiber, Nerve conduit

## Abstract

Multichannel structures in the design of nerve conduits offer potential advantages for regeneration of damaged nerves. However, lack of biochemical cues and electrical stimulation could hamper satisfactory nerve regeneration. The aim of this study was to simultaneously evaluate the effects of topographical, biological, and electrical cues on sciatic nerve regeneration. Accordingly, a series of multichannel nerve conduit was made using longitudinally-aligned laminin-coated poly (lactic-co-glycolic acid) (PLGA)/carbon nanotubes (CNT) nanofibers (NF, mean diameter: 455 ± 362 nm) in the lumen and randomly-oriented polycaprolactone (PCL) NF (mean diameter: 340 ± 200 nm) on the outer surface. In vitro studies revealed that the materials were nontoxic and able to promote cell attachment and proliferation on nanofibers and on fibrin gel. To determine the influence of laminin as biological and CNT as electrical cues on nerve regeneration, either of hollow PCL conduits, PLGA NF-embedded, PLGA/CNT NF-embedded or laminin-coated PLGA/CNT NF-embedded PCL conduits were implanted in rats. A new surgery method was utilized and results were compared with an autograft. The results of motor and sensory tests in addition to histopathological examination of the regenerated nerves demonstrated the formation of nerve fibers in laminin-coated PLGA/CNT NF-embedded PCL conduits. Results suggested that these conduits have the potential to improve sciatic nerve regeneration.

**Figure:**
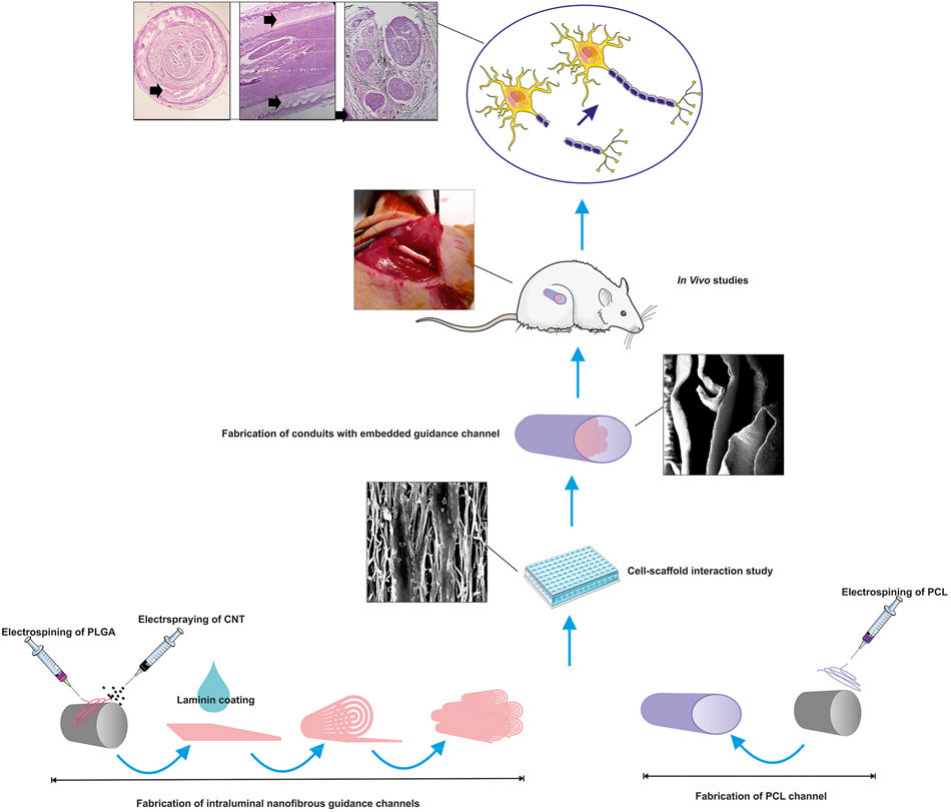
Graphical abstract

Graphical abstract

## Introduction

Nerve injuries impose a huge burden on the healthcare systems all over the world [1]. Although some of the nerve gaps become repaired through the natural mechanisms of the peripheral nervous system (PNS), extensive damages need to be reconstructed surgically [1]. If regeneration does not occur, it could result in neuroma and muscle atrophy [1, 2]. Autologous nerve grafts have been considered as the best standard options for repair [3]. Nevertheless, it may cause some problems in donor site such as tissue morbidity. As an alternative, development of nerve conduits has been considered. These structures are used to provide a similar native extracellular matrix (ECM) environment to bridge the gap and direct the regenerating axons of proximal stump toward the distal one [4, 5]. However, none of the commercially-available nerve conduits act better than standard technique of autologous nerve grafting [5–7]. Therefore, it has prompted designing of new synthetic scaffolds to treat nerve injuries.

Recent developments aimed to design the scaffolds that mimic the native ECM by presenting appropriate biochemical and physical cues [7–12]. The ECM of PNS exhibits special architecture and microenvironment to guide neurite regeneration [4]. As the natural nerve is composed of tubular fascicles, a simple tube cannot replicate the intrinsic characteristics of the natural nerve [13]. Thus, designing the lumen with longitudinally-oriented channels associated with aligned NFs would mimic the bands of Büngner where Schwann cells (SCs) form aligned bands between damaged nerve ends to guide axons [5, 9].

In addition to architectural design, the electrophysiological properties of synthetic conduits can significantly promote cellular responses and differentiation into neural lineage [14, 15]. Recent studies revealed that conduits filled with parallel-aligned conductive fibers facilitate the functionality of regenerating nerves [16] even better than autologous nerve grafts [14, 16]. In order to take advantage of electrical stimulation, scaffolds with different levels of electrical conductance have been designed [14, 16, 17]. Multi-walled carbon nanotube (MWCNT) is an excellent choice to be used as a filler in the scaffolds to improve their mechanical and electrical properties [18, 19]. Also, utilization of bioactive molecules such as laminin, as a primary component of basal lamina, has been suggested to further improve nerve regeneration and neurite outgrowth [2, 14, 20–24].

Previous studies showed that PCL conduits could provide ideal support for different tissue engineering applications [25–28] including axonal regeneration both in wider gaps and longer periods of time that are needed for nerve regeneration in human. According to the properties of the above-mentioned materials, in the present study, a series of multi-channel nanofibrous nerve conduits was fabricated. The outer layer was made of random electrospun polycaprolactone (PCL) NF to provide physical support. The channels were comprised of aligned poly lactic-co-glycolic acid (PLGA) NF as their degradation rate can be engineered to fit particular needs [22]. The structures were characterized and cyto-compatibility of the conduits was evaluated. Finally, the in vivo functionality of nerve conduits was comprehensively investigated using a new and efficient surgery method in a rat model of sciatic injuries.

## Materials and methods

### Materials

MWCNT (-COOH functionalized, diameter of 20–30 nm with a length of 10–30 µm) was purchased from US Research Nanomaterials, USA. PLGA with a copolymer ratio 50:50 (Mw = 50 kDa) and PCL (Mw = 70 kDa) were purchased from Esun Industrial Company, China. Dulbecco’s Modified Eagle’s medium (DMEM), fetal bovine serum (FBS) and penicillin–streptomycin were obtained from Gibco, USA. 3-hydroxytyramine (dopamine) hydrochloride, Engelberth–Holm–Swarm murine sarcoma laminin-1, anti-rabbit IgG-FITC, anti-S100 antibody, bovine serum albumin (BSA), diacetate (4,6-diamidino-2-phenylinolole) (DAPI), MTT reagent, Tris (hydroxymethyl) aminomethane and all solvents such as methanol, ethanol, and 1,1,1,3,3,3-hexafluoro-2-propanol (HFIP) were obtained from Sigma Aldrich, USA and used as received without further purification.

### Fabrication of PLGA/CNT intra-luminal nanofibrous guidance channels

CNT solution (0.5%, w/v) in ethanol and PLGA solution (17%, w/v) in HFIP were separately prepared. The solutions were loaded into two separate plastic syringes. Then, two distinct syringe pumps were utilized to feed the solutions into two 21G nozzles placed on opposite sides of the rotating drum (2500 rpm) and then, parameters of PLGA electrospinning and CNT electro-spraying were set according to the Table 1. The PLGA/CNT NF were collected on the aluminum foil wrapped around a rotating drum.

**Table 1:** Parameters of PLGA electrospinning and CNT electro-spraying

Parameters	PLGA syringe	CNT syringe
Feeding rate (ml/h)	0.7	1.4
Distance from drum (cm)	17	10
Voltage (kV)	17	14

### Laminin-coating on intra-luminal nanofibrous guidance channels

The scaffolds were coated with laminin based on our previous report [17]. In brief, PLGA/CNT scaffolds were immersed in dopamine (2 mg/ml) solution prepared from 10 mM Tris-HCl buffer, pH 8.5, and shaken for 1 h at room temperature [29]. Then polydopamine- coated PLGA/CNT scaffolds were washed with DI water three times to remove the unbound dopamine. The scaffolds were incubated with laminin (100 µg/mL) overnight at 4 °C, followed by washing with DI water [30, 31].

### Fabrication of PCL conduits with intra-luminal nanofibrous channels

A 12% w/v solution of PCL was prepared in chloroform/methanol (70/30, v/v) with stirring for 4 h. The solution was electrospun by loading into a 5 ml syringe equipped with a blunt-end 21G needle. Fibers were electrospun onto a rotating mandrel (2 mm diameter) with a rotation rate of 1000 rpm for conduit manufacture. The spinning conditions for polymer solution were set according to Table 2.

**Table 2:** Parameters of PCL conduit electrospinning

No.	Voltage (kV)	Tip-drum distance (cm)	Feed rate (ml/h)
1	17	10	1
2	17	13	1
3	17	16	1
4	17	16	0.7
5	14	13	1
6	11	13	1

Electrospun PCL fibers on a rotating mandrel were collected as a tube wall. Then, the stainless steel rod was removed and the PCL nanofibrous tube was obtained. After incubating in a vacuum oven overnight, the laminin-coated (PLGA/CNT-lam) and un-coated PLGA/CNT membranes were cut into 1 cm^2^, rolled, and placed in the PCL nanofibrous tube.

### Characterization of surface morphology

The surface morphology of the PLGA/CNT-lam scaffolds, PCL conduits, and the whole structure of conduits were analyzed by scanning electron microscopy (SEM, XL 30, Philips, USA). DiameterJ and OrientationJ, which are ImageJ plugins were used to estimate the diameter and orientation of the PLGA/CNT-lam NF.

### Mechanical properties

To measure suture retention strength of conduits, following the ANSI/AAMI/ISO 7198:1998/2001/R2010 recommendations. One end of conduits was immobilized by the bottom stage clamp of the testing machine (Zwick/Roell, Z020, Germany), and the other end was connected to the movable top clamp by a single loop of 6–0 nylon suture inserted diametrically through the walls 2 mm from the edge of each conduit and tied to form a loop. The sutures were pulled out at a constant rate of 50 mm/min at room temperature (25 °C) working with a load cell of 100 N until it tore through the sample. Maximum force was recorded [32].

### Analyzing surface electrical resistance

The electrical resistance of PLGA/CNT and PLGA/CNT-lam scaffolds were analyzed by a digital four-point probe apparatus (5450/5451, ADCMT, japan). In this method, a corresponding electrical current was obtained when providing a voltage for samples cut in square shape. One pair of probes was used for the current injection while the other probes were used for the voltage measurement.

### Cell–scaffold interaction studies

#### In vitro SC culture

To study cells’ viability and their behavior on scaffolds, SCs were used. They were isolated from the sciatic nerves of adult male Wistar rats purchased from Pasteur Institute, Tehran, Iran according to Terraf et al. [33]. The cells were cultured in a 25 ml cell culture flask in DMEM, supplemented with 10% FBS and 1% penicillin/streptomycin antibiotics at 37 °C in a humidified 5% CO_2_ incubator.

#### Cell proliferation assay

The PLGA/CNT-lam and PCL scaffolds were placed in a 24-well plate and fixed with plastic rings. Then they were sterilized by adding 70% ethanol and exposing it to ultraviolet light for 30 min, followed by washing with PBS three times [34]. After the confluence was sufficient, SCs were detached by trypsin/EDTA, counted, and seeded on the scaffolds with a density of 1.0 × 10^4^ cells/well. Cell proliferation on the scaffolds was analyzed using MTT assay after 1, 3, and 5 days of cell culture and evaluated in comparison with control well (TCP) containing only SCs lack of NF. At mentioned time points, MTT solution was added, incubated in darkness for 4 h at 37 °C, and then, replaced by dimethyl sulfoxide to dissolve the formazan crystals and finally absorbance was read at 570 nm by a plate reader.

#### Cell attachment and morphology analysis of SCs on the scaffolds

After 5 days of SCs incubation on the scaffolds, they were washed with PBS and processed with 4% paraformaldehyde to fix the cells on the scaffolds. After rinsing the samples with PBS, the samples were dehydrated using gradient ethanol (30–100%) and then, dried at room temperature. To evaluate the cells’ morphology and orientation, samples were gold-sputtered and observed by SEM. In addition, the cell-scaffold constructs were characterized by antibodies against S100 protein-specific marker for SCs. The samples were rinsed with PBS and fixed 4% formaldehyde solution for 30 min, followed by permeation with 0.1% TritonX-100 for 1 min. Non-specific labeling was blocked by incubating scaffolds in 2% BSA solution for 1 h and then samples were immersed in mouse anti-S100 (diluted at 1:1000) overnight. Subsequently, specimens were washed with PBS and incubated in FITC-conjugated goat anti-mouse secondary antibody (diluted at 1:100) for 1 h. Furthermore, the cells on samples were counterstained with 1 µg/ml DAPI for 30 min and immunofluorescence imaging was performed using the fluorescence microscope [35].

#### SCs culture in fibrin gel

SCs were re-suspended in 3 mg ml^−1^ fibrinogen solution at 1 × 10^4^ cells/ml. 90 μl of fibrinogen/cell solution was added to 10 μl thrombin solution (2 mg ml^−1^) and pipetted into the end of each segment of conduits (3 mm length). The fibrin changed into the gel form and then, DMEM with 10% FBS and 1% antibiotic was added to each guide in a 48-well plate. Cells were cultured for 48 h in a 37 °C incubator at 5% carbon dioxide.

#### Cell infiltration in nerve conduits

To evaluate cell infiltration, samples were fixed with a combination of 2% paraformaldehyde and 2.5% glutaraldehyde for 90 min. followed by dehydration with gradient ethanol (30–100%). Finally, they were placed on SEM stubs, gold-sputtered and imaged with FESEM (FESEM, Zeiss SUPRA 55-VP, Oberkochen, Germany).

### In vivo studies

Thirty healthy adult male Wistar rats (250–270 g) were purchased from Pasteur Institute (Tehran, Iran). Animal experiments were approved by the ethical committee of Tehran University of Medical Sciences and were performed according to the university’s guidelines. The rats were randomly divided into 6 groups of five: negative control (with injury but without surgical intervention), hollow PCL conduit, PCL conduit with PLGA membranes (PCL-PLGA), PCL conduit with PLGA/CNT membranes (PCL-PLGA/CNT), PCL conduit with PLGA/CNT-lam membranes (PCL-PLGA/CNT-lam) and autograft. For surgical procedure, the animals were anesthetized by intra-peritoneal injection of ketamine (30 mg/kg) and xylazine (10 mg/kg).

**Fig. 1:**
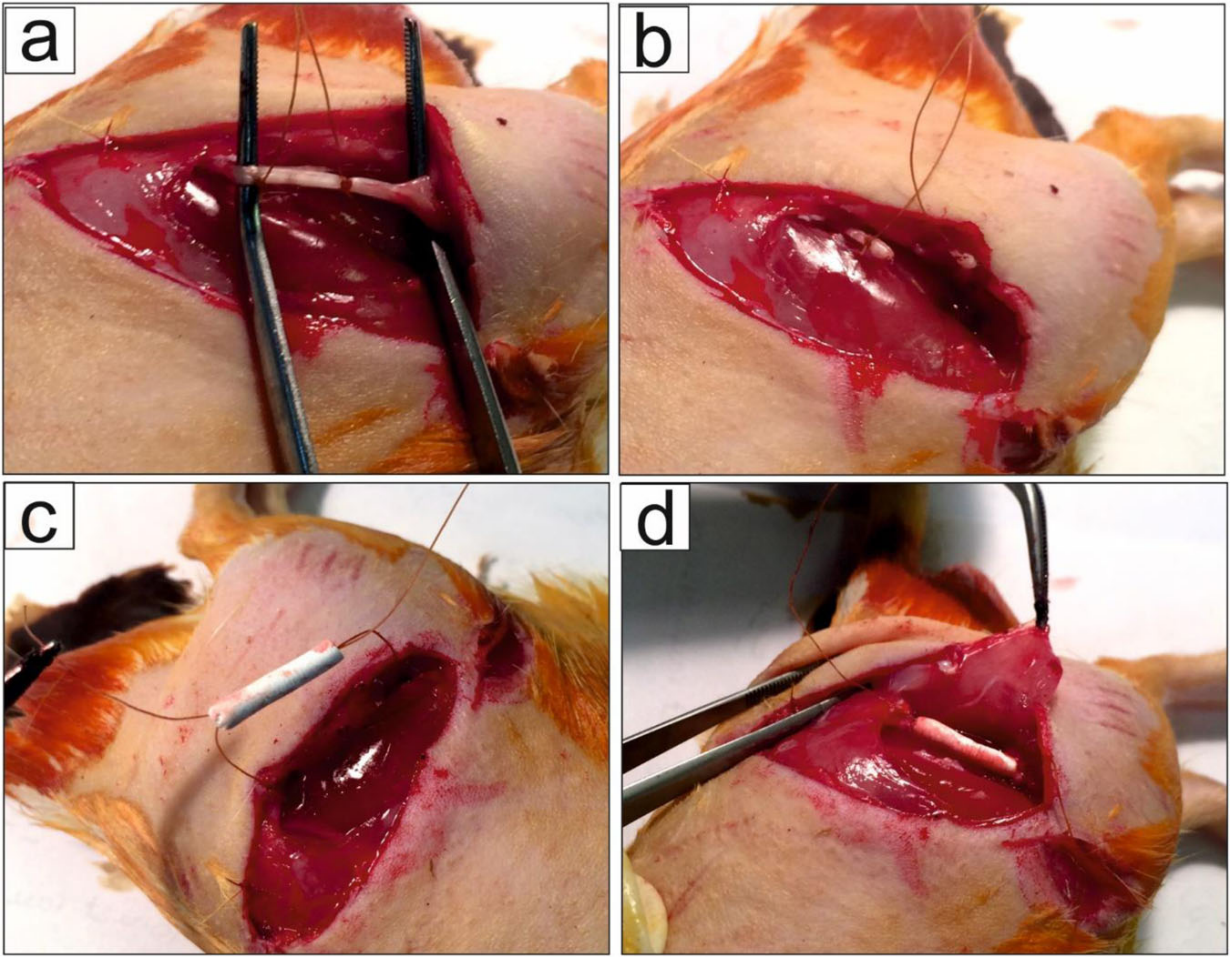
Surgical implantation of a PCL-PLGA/CNT-lam conduit bridging a 10 mm sciatic nerve defect in rats (**a**, **b**), the sciatic nerve was fixed with two sutures **c**, **d**. The suture strings were transmitted in the conduit and pulled out in transverse directions

To expose sciatic nerve, a skin incision was made at the left lower limb of the animal and the sciatic nerve was fixed with two sutures (Fig. 1a) before severing into proximal and distal segments at the center of the right thigh. Both ends of proximal and distal stumps were fixed with one suture using 6–0 nylon sutures (Fig. 1b). The suture strings were transmitted in the conduit channel and pulled out in transverse directions and allowed the nerve stumps to insert into the conduit to a depth of about 3 mm (Fig. 1c, d), leaving a 10 mm gap between the stumps. Finally, they were tied together after coming out of the conduit. Then, the skin was closed with 3–0 nylon. In the nerve autograft group, the nerve defect was bridged with the resected nerve segment which was reversed and anastomosed to the proximal and distal stumps. In the negative control group, there was no bridge to connect the two stumps. All the implantation procedures were conducted under microscope using microscopy instruments. After surgery, rats were housed and fed regularly and also, their activities were monitored.

#### Walking-foot-print analysis

Walking track analysis were performed 6 and 12 weeks post-surgery [36]. In this test, the rat footprints were recorded for the analysis of the sciatic functional index (SFI). The rat hind paws were soaked in different color inks and they were placed inside a wooden walking alley (43 cm length, 8.70 cm width, and 5.50 cm height) covered with a millimeter paper and ended with a darkened goal box. SFI was calculated by the following equation:

$$\begin{array}{l}SFI = - 38.30 \times \left[ {\frac{{OPL - NPL}}{{NPL}}} \right] + 109.5 \times \left[ {\frac{{OTS - NTS}}{{NTS}}} \right]\\\qquad\quad + 13.30 \times \left[ {\frac{{OIT - NIT}}{{NIT}}} \right] - 8.80\end{array}$$

where PL is the distance from the heel to the top of the third toe, TS is the distance between the first and the fifth toe and IT is the distance from the second to the fourth toe. In addition, N and O related to the non-operated and operated foot and index of 0 indicated the normal function while −100 represented total impairment.

#### Nerve conduction test

Twelve weeks post-surgery, to evaluate nerve regeneration, compound muscle action potential (CMAP) amplitudes of the sciatic nerves were evaluated [36]. The animals were anesthetized by injection of ketamine/xylazine and the sciatic nerve proximal to the site of the injury was exposed and stimulated with an electric stimulus (3–5 mA) by means of needle electrodes. To remove any probable interference, a ground electrode was located inside the muscle adjacent to the nerve. The responses were recorded from the needle and cap electrodes (filtering frequency of 10 Hz to 10 kHz, sensitivity of 2 mV/division, and sweep speed of 1 ms/division) by means of an electromyographic recorder (Negarandishegan, Iran).

#### Gastrocnemius muscle wet weight loss

Twelve weeks post-surgery, weight loss of gastrocnemius muscles of the rats was determined [36]. To do this, rats were sacrificed and the posterior gastrocnemius muscles on the injured and uninjured hind limbs were weighed and the wet weight-loss of gastrocnemius muscles was calculated by the following equation:

$$\begin{array}{l}Gastrocnemius\,muscle\,wet\,weight - loss\left( {{{\mathrm{\% }}}} \right)\\ \displaystyle = \left( {1 - \frac{{Wet\,weight\,of\,the\,muscle\,on\,the\,injured\,side}}{{Wet\,weight\,of\,the\,muscle\,on\,the\,uninjured\,side}}} \right) \ast 100\end{array}$$

#### Functional assessment of sensory recovery (Hot plate latency test)

Twelve weeks post-surgery, the rats were evaluated for hot plate latency (HPL) by placing their injured limbs on the center of a hot plate (56 ˚C) and recording the time until they reacted (by jumping or licking their paws). The rats were removed from the plate after their reaction. The cut-off time for test was set at 12 s.

#### Histopathological examination

The animals were euthanized 12 weeks post-surgery and the sciatic nerve in the place of the graft was dissected out and fixed with 10% neutral buffered formalin (NBF, PH. 7.26) for 48 h. The harvested tissue samples were processed, embedded in paraffin blocks, and cut into 5 mm thick sections. For the nerve specimens, transverse (proximal and distal parts) and longitudinal (middle part) sections were obtained from the sciatic nerve (cutting area). All nerve sections were made in duplicate: the first one was stained with haematoxylin and eosin (H&E) and the second one by luxol fast blue (LFB). Finally, histological slides were evaluated by the independent reviewer, using light microscopy (Olympus BX51; Olympus, Tokyo, Japan).

### Statistical methods

All data in this study are reported as mean ± standard deviation. One-way analysis of variance was conducted and followed by Tukey’s post hoc. The level of statistical significance is defined as *(*p* < 0.05), **(*p* < 0.01), ***(*p* < 0.001), ****(*p* < 0.0001). Each parameter was conducted at least with three samples.

## Results

### Characterizations of the intraluminal PLGA/CNT-lam and PCL conduit

**Fig. 2:**
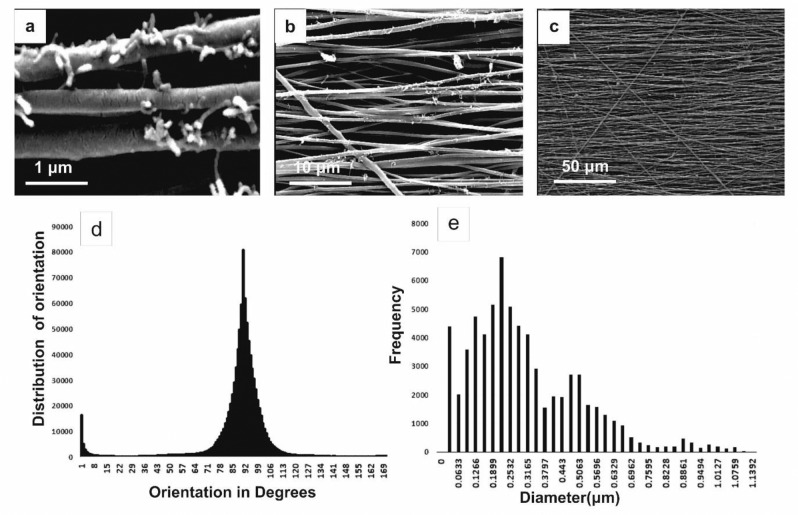
FESEM micrograph of PLGA/CNT-lam scaffolds (**a**–**c**, scale bars: 1, 10, and 50 µm), distribution of fiber orientations (**d**) and fibers diameters **e**. NF of PLGA/CNT-PD-lam scaffolds was aligned with the average diameter of 455 ± 362 nm

Electrospun PLGA/CNT scaffolds were prepared as intraluminal guidance channels. Thereafter, our previously-reported method was applied to coat the electrospun PLGA/CNT scaffolds with laminin by means of PD layer [17]. As shown in Fig. 2, PLGA/CNT-lam scaffolds were fabricated as longitudinally aligned nanofibrous guides with an average diameter of 455 ± 362 nm. The SEM results revealed that the fibers were aligned and continuous without breaking under the rotation speed of 2500 rpm and were covered with MWCNTs partially.

**Fig. 3:**
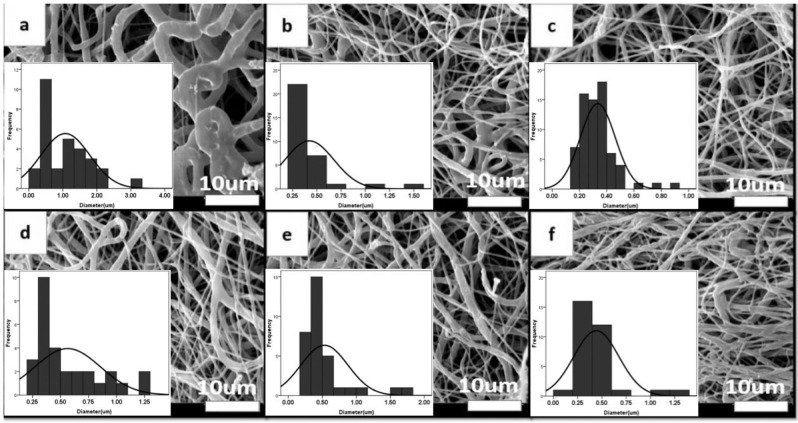
SEM micrograph and fiber distribution diagrams of nanofibrous PCL conduits with different spinning conditions according to the parameters of Table 2 (1–6) (**a**–**f**, scale bars: 10 μm). The best result is shown in micrograph **c** related to sample 3 in Table 2 could lead to PCL conduit with mean diameter of 340 ± 200 nm

Three electrospinning parameters namely applied voltage, needle-collector distance, and feeding rate have been varied to investigate their influences on the PCL fiber morphology. It was found that when the distance between nozzle and drum was 10 cm, most of the fibers between the nozzle and drum merged (Fig. 3a), increasing the distance to 13 and 16 cm reduced the number of merged fibers and the mean fiber diameter decreased (Fig. 3b, c). Another parameter, the feeding rate, influenced the thickness of channel in a way that reducing the feeding rate to 0.7 ml/h led to the formation of channel with inadequate thickness (50 µm) (Fig. 3d). When the applied voltage was lower than 17 kV, jet could not form well and the solution sometimes dripped from the syringe tip (Fig. 3e, f) [37]. Eventually, the parameters of sample 3 were selected as the best condition to fabricate channel with relatively uniform NF. Diameters of the obtained NF were 340 ± 200 nm, and porosity percentage was ~52%. Electrospun PCL fibers that were collected onto a 2 mm-diameter rotating mandrel formed the hollow channel with uniform wall thickness of about 200–220 μm, length of 17 mm, and inner diameter of 1.8 mm. It is previously proved that scaffolds of 100–200 μm provide sufficient diffusion for nutrient exchange and waste transport for nerve repair [38, 39]. In addition, there were some pores among NF (<10 μm) which can facilitate the exchange of different nutrients and metabolic products through the conduit wall [40].

Suture retention test was performed to assess the mechanical strength of the conduits. The suture retention strength of nanofibrous conduits with random alignment was 3.2 ± 0.2 N which is favorable from a surgical standpoint according to another study [41]. Also, we have previously reported the mechanical properties of structures related to the embedded nanofibers inside the conduits [42].

**Fig. 4:**
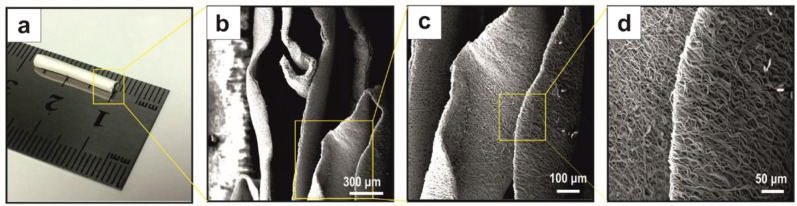
An image of the nanofibrous conduit (**a**) and the cross-sections of PCL-PLGA/CNT-lam conduit (**b**–**d**, scale bars: 300, 100, and 50 μm). Aligned PLGA/CNT-lam fibers were successfully embedded within the PCL channel

As shown in Fig. 4, PLGA/CNT-lam sheets with aligned fibers were rolled and placed in the nanofibrous PCL tube to direct neurite growth during regeneration.

SEM image analyses of the conduit cross-sections revealed that the aligned functionalized electrospun PLGA/CNT fibers were successfully embedded within the PCL channel with random orientation (Fig. 4).

As electrical cue is one of the important factors for neural regeneration [43], the conductivity of the PLGA/CNT, PLGA/CNT-Lam scaffolds was evaluated by the four-point probe method, and the values were measured as 0.17 and 0.11 S/cm. The measurements revealed that a very thin coating layer could not significantly change the surface conductivity of the scaffolds. According to the existing concerns about toxicity and degradation of CNTs used in biomaterials for neuroengineering, we applied electro-spraying CNT with the possible minimum concentration of CNTs to fabricate electro-conductive scaffolds [44].

### In vitro study of the SCs

#### Cell viability assay

**Fig. 5:**
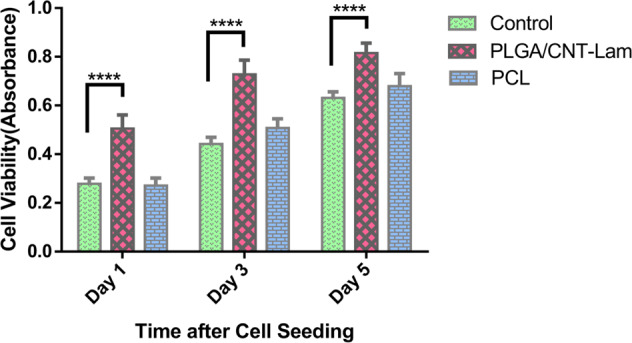
Cell viability of the SCs cultured on PCL and PLGA/CNT-lam scaffolds. The chart illustrates the higher cell viability on PLGA/CNT-lam compared to TCP

Here, we evaluated SCs viability on nerve conduits and seeded them onto the intraluminal guides and PCL channel for 1, 3, and 5 days. The proliferation of the SCs on the scaffolds was compared to control group (TCP) in all pre-defined times. According to Fig. 5, the cell viability on PLGA/CNT-lam was higher than that on TCP and these modified scaffolds had stronger effect on the improvement in cell proliferation.

#### Morphology of the SCs

**Fig. 6:**
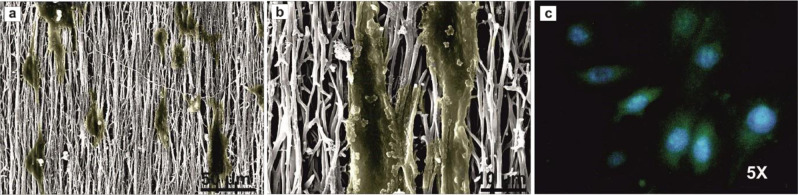
SEM images with different magnifications (**a**: 50 µm, **b**: 10 µm) and immunofluorescence staining (**c**) of the SCs cultured on the aligned PLGA/CNT-lam NF (Magnification 5×)

Five days after seeding of the SCs on the aligned PLGA/CN-Lam nanofibrous scaffolds, SEM analyses were done to observe cell attachments and morphologies (Fig. 6a, b). Furthermore, the morphological evaluation of cell proliferation was also performed by fluorescence staining (Fig. 6c).

#### Cell infiltration

**Fig. 7:**
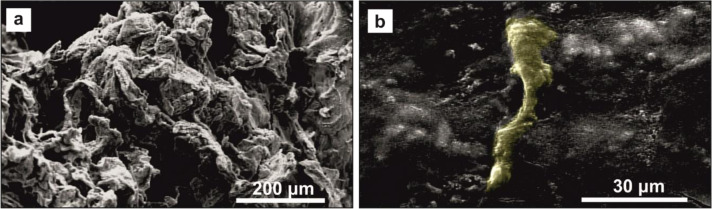
FESEM image of fibrin gel before (**a**) and after (**b**) SCs culture. The porous microstructure of hydrogel could facilitate SCs infiltration

Figure 7 demonstrates the FESEM images of fibrin gel before and after cell culture in the conduit. It should be considered that the gel porosity in Fig. 7a was better maintained due to freeze-drying step. The attachment and cell–scaffold interactions were observed after 48 h of cell seeding (Fig. 7b).

### In vivo study

All animals tolerated anesthesia without complications. During in vivo study, different tests were performed to assess functional and sensory properties of rats 12 weeks post-surgery. While nerve autografts are the current gold standard in the field of peripheral nerve surgery, all groups were compared with them.

#### Walking-foot-print analysis (SFI)

**Fig. 8:**
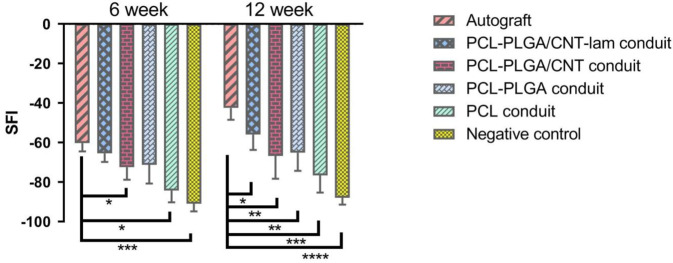
Sciatic nerve functions of rats as a function of implantation time. The statistical analyses were done by comparing the SFI values of each group in a specific time-point to the autograft. The SFI value for PCL-PLGA/CNT-lam group was significantly (*p* ≤ 0.05) higher than that of the PCL conduits at the end of the 12th week

Figure 8 demonstrates the average SFI values for function of the injured feet in different groups at 6 and 12 weeks post-surgery. In the PCL-PLGA/CNT-lam group, the SFI value increased from −65.46 ± 4.3 at week 6 to −55 ± 78 at week 12. The analysis showed better functional recovery results in the autograft group that were −60.28 ± 4.2 and −42.43 ± 6.02 at 6 and 12 weeks, respectively. Findings indicated that there was a significant (*p* ≤ 0.05) difference between autograft and PCL-PLGA/CNT-lam group and none of the conduits could act as well as the autografts (Fig. 8). Furthermore, the data of conduits with embedded nanofibrous membranes did not differ significantly from each other and they could recover neural functionality to a certain degree.

#### Electrophysiological analysis

**Fig. 9:**
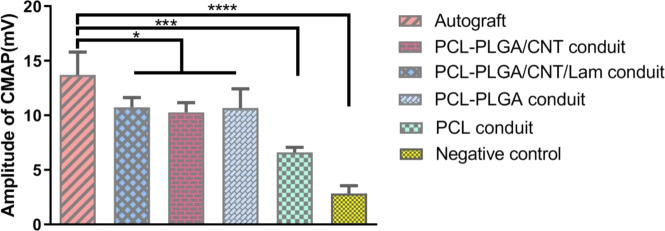
Electrophysiological analysis of the regenerated nerves 12 weeks post-operation. The statistical analysis was carried out by comparing the amplitude of CMAP values of each group to the autograft. The peak CMAP value in the PCL-PLGA/CNT-lam group was much higher than the values for the PCL group and negative control

Motor nerve conduction study was performed by electrical stimulation of damaged sciatic nerve and recording CMAP as a response of gastrocnemius muscle supplied by that nerve to evaluate the muscle reinnervation. The peak CMAP value in the PCL-PLGA/CNT-lam group (10.75 ± 0.88 mV) was much higher than that of the PCL group (6.6 ± 0.47 mV) but recovery of the transected sciatic nerve was still poorer than that of the autograft group (CMAP 13.73 ± 2 mV) at 12 weeks post-surgery. As shown in Fig. 9, other conduits with embedded nanofibrous membranes did not differ significantly from each other but they had higher CMAP amplitude than PCL conduit and negative control.

#### Assessment of gastrocnemius muscle wet weight

**Fig. 10:**
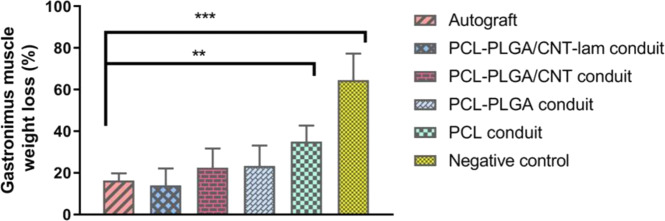
Assessment of gastrocnemius muscle wet weight 12 weeks post-surgery. PCL-PLGA/CNT-lam conduits and autograft have similar effects on preventing muscle atrophy

Untreated nerve injury can lead to muscle denervation, atrophy, and weight loss [45]. Gastrocnemius muscles were innervated by branches of sciatic nerve. Thus, its weight loss is a parameter used to indirectly assess the related motor neuron defects. As shown in Fig. 10, there were no statistically significant differences between the autograft and groups with nanofibrous embedded conduits or between every two groups (*p* > 0.05).

#### Hot plate latency (HPL) test

**Fig. 11:**
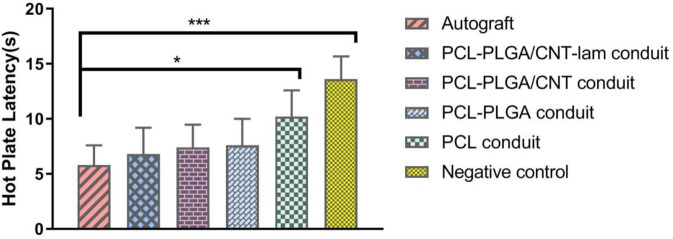
Hot plate jumping response of rats 12 weeks post-surgery. The withdrawal response displayed much better sensory recovery results in PCL-PLGA/CNT-lam group than negative control

As shown in Fig. 11, 12 weeks after injury, rats in the negative control group could not withdraw their paws from the hot plate within 12 s. The withdrawal response displayed much better sensory recovery results in the autograft (5.8 ± 1.8 s) and PCL-PLGA/CNT-lam group (6.81 ± 2.38 s). Furthermore, there were no statistically significant differences between the HPL times of groups with nanofibrous embedded conduits, proposing that these conduits can help in regeneration of the sensory axons across the gap. The latency time for the hollow PCL group was recorded to be 10.2 ± 2.37 s, which was significantly smaller than that of the negative control group (13.6 ± 2.07 s).

#### Histological changes in injured sciatic nerve

**Fig. 12:**
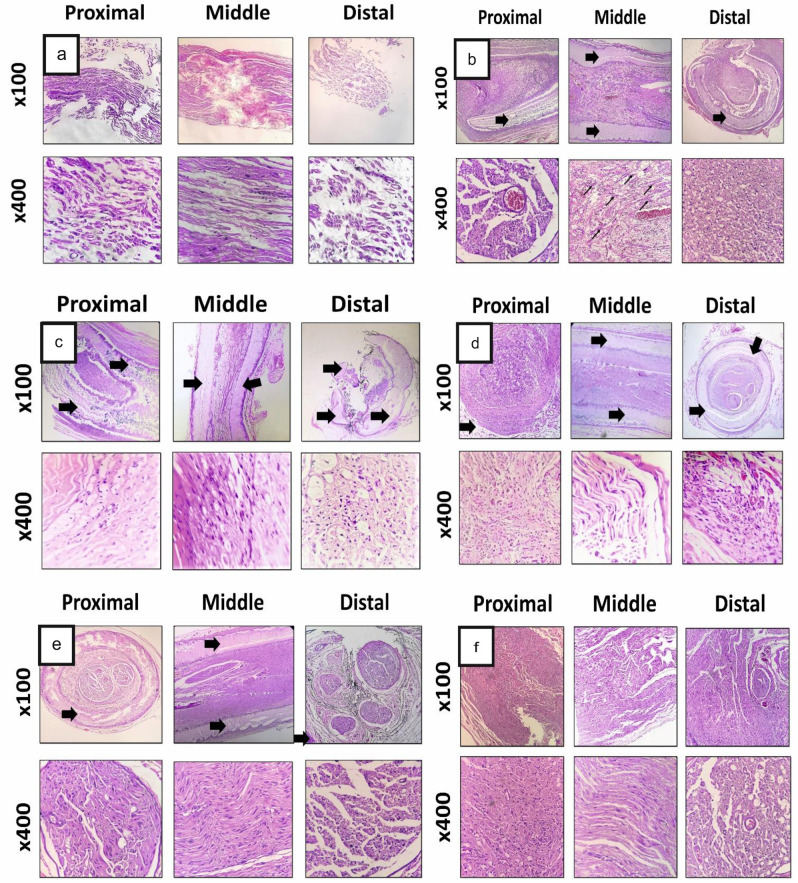
Histopathological examination of the sciatic nerve cross-sections stained by hematoxylin-eosin (H&E) at the end of 12th week post-surgery. Negative control group (**a**), PCL group (**b**), PCL-PLGA group (**c**), PCL-PLGA/CNT group (**d**), PCL-PLGA/CNT-lam group (**e**), and autograft group (**f**). Thin arrows: regeneration of nerve fibers, big arrows: remnant conduit (magnification: 100× and 400×)

The arrangement of sciatic nerve fibers in the negative control group was disrupted and they had swollen or missing axons with various degrees of vacuolation. In addition, multiple signs of nerve damage were observed, including irregular distribution and variable thickness of myelin sheath, degeneration of fibers, axonal disintegration, and notable edema of the nerve fibers. The number of axons was significantly decreased (especially in distal area) in comparison to other treatments (Fig. 12a). In other groups with nerve conduits, the tubular PCL structures could be differentiated from regenerated tissues within them. They were shown with arrows both in cross-sectional and, longitudinal-sections of the regenerated nerves. In the PCL group, the arrangement of the sciatic nerve fibers was acceptable, however, in the middle part, the bridging process was incomplete and the number of axons was significantly decreased compared to the proximal part. Moreover, in distal part, nerves had swollen or missing axons with various degrees of vacuolation (Fig. 12b). In the PCL-PLGA group, the nerve fibers were symmetrically distributed and thickness of myelin sheath was normal. The biocompatibility of this conduit was completely acceptable and this finding was confirmed in all samples. There was no sign of inflammatory responses in this sample due to the implanted conduit and using nylon suture (Fig. 12c).

In the PCL-PLGA/CNT group, the histopathological analysis showed uniform myelin sheaths and normal axonal structure (Fig. 12d). There was no sign of fibrosis and the nerve fibers were free in proximal, middle, and distal parts.

**Fig. 13:**
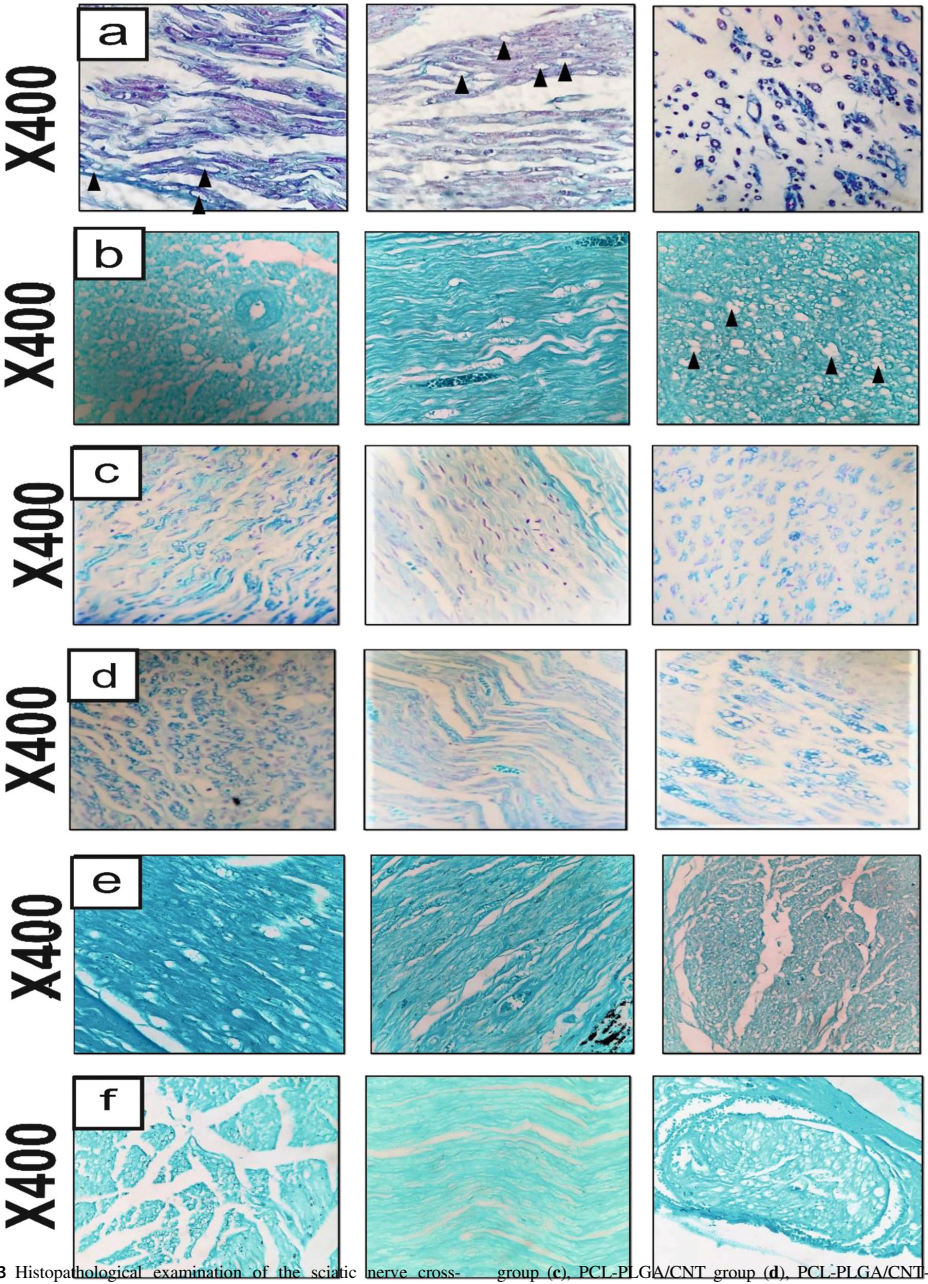
Histopathological examination of the sciatic nerve cross-sections stained by and Luxol fast blue (LFB) at the end of 12th week post-surgery. Negative control group (**a**), PCL group (**b**), PCL-PLGA group (**c**), PCL-PLGA/CNT group (**d**), PCL-PLGA/CNT-lam group (**e**), and autograft group (**f**). Arrowheads: vacuolation, (magnification: 400×)

In PCL-PLGA/CNT-lam group, there were no significant histopathological changes in sciatic nerve and the bridging process (regeneration) was complete. However, the number of axons was decreased in distal part when compared to the proximal and middle part of sciatic nerve (Fig. 12e). In addition to the tubular PCL structure, the black remnants of PLGA/CNT-lam intra-luminal nanofibrous scaffolds were evident in this group. In the autograft group, although the arrangement of sciatic nerve fibers in proximal parts was disrupted, this nerve was intact in the middle and distal parts (Fig. 12f). These results are in agreement with previous studies reported most of the nerve fibers can return to the normal state and that uniform myelin sheaths can form after 12 weeks [36]. The findings from H & E images were also confirmed with LFB staining which stains the myelin blue (Fig. 13).

The overall results demonstrated that the incorporation of membranes with topological cues was important for nerve regeneration and presence of nylon suture did not induce inflammatory reaction.

## Discussion

In the present study, fabrication of aligned nanofibrous membranes has been considered. Previous studies reported that aligned electrospun fibrous scaffolds of 400–600 nm in diameter have been shown to orient dorsal root ganglion alignment and SC migration in vitro [46]. Another study revealed that nanofibrous scaffolds can support the growth and migration of cultured neural cells by means of an increase in cell spreading on fibers diameters of 400 nm and 800 nm [6, 47]. Orientation of the fibers could be modulated by the collector rotation speed and faster rotation speed resulted in more uniform fibers. However, it becomes limited by fiber breakage that could happen during the electrospinning on collector with a very fast rotation speed [48]. Indeed, unidirectional fiber alignment can facilitate axon pathfinding and increase the speed of nerve tissue regeneration [49, 50].

PLGA was selected to be the intraluminal guidance material as it has a faster degradation rate. This allows the guidance to degrade after the axons regenerate. As the purpose of the intra-luminal guidance is to direct the axon growth cone across the gap, once the first axons have bridged the gap, the presence of intraluminal guidance is no longer necessary and its degradation provides more space to fill by the native ECM [51].

The obtained fibers were used to resemble the natural nerve structure and provide topographical, biological, and electrical cues of natural nerve tissues which were expected to play critical roles to guide and improve axonal growth. The complementary effects of these three factors can lead to more complete nerve regeneration by acting on different subcategories of re-growing fibers. Thus, our design provides a three-dimensional construct with open channels to guide and stimulate neurons and SCs between the aligned functionalized fibrous substrates. The PCL channel which provides a barrier between the healing nerve and surrounding media was fabricated from nanofibers with relatively non-uniform distribution. Previous studies revealed that the electrospun solution condition (including viscosity, charge density and shear stress) and solvent vapor pressure can simultaneously affect the fiber diameter distribution [52]. Another parameter which can affect fiber distribution is collector rotation. The impact of airflow during rotation can cause fluctuation in fiber stretching and disturb the jet drawing [53].

It is reported that the scaffold with electro-conductivity of 3.90 × 10^−4^ S/cm could facilitate electrical stimulation to promote axonal regrowth [44]. The conductive scaffolds can transmit the self-originated electric stimulation between the nerve cells which can be the basis for transmission of electrical signals in the nerves and regeneration of the peripheral nerves [54].

In our previous work, PC12 cells have been used to study the cell viability and cell attachment of PLGA, PLGA/CNT, and PLGA/CNT-lam scaffolds. Results revealed that all of the scaffolds increased cell viability as time rises and laminin-coated scaffold could promote neurite outgrowth more than others [17].

As SCs migrate to the injury site after nerve injury and align to form bands of Büngner to support axonal regrowth, the biocompatibility of the conduit with SCs has an important influence on the final result [44]. According to Fig. 6a, b, the SCs excellently adhered to the scaffolds and extended along the main axis of the fibers which was due to the significant effects of aligned nano-topography [55]. Also, as SCs guide regenerating axons, this nano-topography can promote the oriented growth of the regenerating axons. Regarding our previous study, these aligned scaffolds could guide the growth of new axons in PC12 cells, as well [17].

As mentioned above, aligned NFs would mimic the bands of Büngner where SCs form aligned bands between damaged nerve ends to guide regenerated axons via providing cell attachment sites and topographical guidance. Also, in vitro studies were performed using fibrin-loaded gels. It is known that fibrin gel formation begins spontaneously after when nerve damages occur in vivo. The longitudinally-oriented fibrin structures could conduct proliferation and migration of SCs and help axonal regrowth to accelerate nerve regeneration [56]. Therefore, to mimic the conditions in vitro, SCs were seeded on fibrin gel in our study. The results revealed that the cell could infiltrate into the fibrin 3D networks and interact with scaffolds in vitro. In addition, in vivo studies were done and histopathological examination results (Figs. 12e and 13e) confirmed the successful formation of nerve fibers. It is noteworthy to mention that the aligned topography of the synthetic nanofibers having PLGA/CNT would help aligned direction of the neural cells and the direction of axonal regrowth.

At present, a majority of studies examined the effect of conductive CNT-containing scaffolds only in vitro on different cells such as PC12 or SCs [8, 42, 57]. However, animal studies would shed light on the functionality of the prepared conduits in vivo. Walking-foot-print analysis was used as a gold standard technique to determine functional recovery following injury to the sciatic nerve. Functional nerve regeneration was assessed by SFI. The results are in agreement with Jing et al. findings about SFI values that the presence of aligned conductive NF in the conduit lumen could increase the performance of the conduit by providing a more effective micro-environment for guiding nerve regeneration [14]. Comparing the SFI values 12 weeks post-operation revealed that the presence of CNT and laminin coating could simultaneously increase motor recovery of injured sciatic nerve and the presence of each factor alone cannot be sufficient. Salehi et al. demonstrated that the presence of protein and MWCNT in the PLA conduit structure as hollow tube cannot improve functional nerve regeneration notably and the SFI value for the rats with these conduits was −67.86 ± 0.58 at the end of 14th weeks [36]. Altogether, it can be concluded that not only the type of materials used in the conduit is important but also the conduit design and the positions of the used materials have great importance.

The results also demonstrated that conductivity and laminin coating of conduits could not affect muscle reinnervation as much as embedding nanofibrous membrane in the conduit. There have been many contradictory reports on the effect of electroconductivity of conduits on nerve conduction [58]. Some of them reported that CMAP was significantly increased when the conduit was conductive and the reinnervation of the muscle was improved as a result of the CNT interfacing [14, 58]. Others reported that CNTs did not have a role in improving and restoring nerve signal conduction [36, 59]. Comparing the results of previous studies revealed that in none of the conduits [14, 36, 58], presence of CNT could not increase the nerve conduction as much as autograft which is consistent with our findings.

According to Fig. 10, there were no statistically significant differences between the autograft and groups with nanofibrous embedded conduits or between each two groups (*p* > 0.05), suggesting that the nanofibrous embedded conduits and autograft have similar effects on preventing muscle atrophy. In other words, regaining muscle weight after implantation of these conduits can be known as indicative of recovery [60]. Comparisons of other groups revealed that even though PCL conduits could increase muscle reinnervation, they could not stop muscle atrophy as efficiently as autografts. Our results are in consistence with previous studies which reported that the muscle reinnervation of conduits with intra-luminal guidance channels was similar to that of the autograft group and groups with added biomolecules demonstrated better performance than autograft group. It suggested that introducing intra-luminal guidance channels has potential to encourage muscle regeneration [39].

To evaluate the sensory recovery, hot plate test was used to assess the pain responses [61]. The first response, licking as a rapid response to thermal stimuli, is known as a direct indicator of nociceptive threshold. Even though jumping is a more elaborated response, with latency, measuring the HPL is used to evaluate the nociceptive function [62]. Comparing HPL times between hollow PCL conduits and embedded conduits revealed that improved recovery of a heat response could be attributed to the enhanced regeneration of the accurate population of sensory axons by means of inner aligned nanofibrous sheets.

Further, our results are in consistent with previous studies that reported PCL conduits conserved their structure 14 weeks post implantation even though breakage might happen [63]. Also, incorporating longitudinally-aligned nanofibrous intra-luminal guidance channels could improve nerve regeneration and confirmed previous findings that reported topographical cues in multi-channels conduit supported SCs migration and axon extensions in vivo [39]. Moreover, our results appeared similar to the recent studies investigated the use of CNT incorporated conduits in vivo which lead to nerve regeneration promotion by accelerating the electrical signal conduction without any toxic effect [36, 44].

We have previously reported that after 1 month of degradation, the PLGA, PLGA/CNT, and PLGA/CNT-lam scaffolds were degraded partially and lost about 20% of their mass [17]. In the present study, these scaffolds were used as intraluminal guide because they could guide axons during the first week and then gradually degrade to generate enough space for nerve regeneration and avoid probable nerve compression. These results are in agreement with previously published studies [64].

The histopathological condition of the PCL-PLGA/CNT/lam group was similar to that of those in the autograft group. It suggests that the PCL-PLGA/CNT/lam conduit could provide the best environment for sciatic nerve regeneration among these four nerve conduits which is consistence with previous reports [65]. Wu et al. developed multi-channel nerve conduits with three-dimensional laminin-coated PLGA yarns and demonstrated that the yarn structure significantly supported SCs proliferation and the laminin-coating cues induced SCs migration [65].

## Conclusion

In this study, we successfully examined a simultaneous effect of biological, topographical, and electrical cues in the form of PCL-PLGA/CNT-lam conduit for the regeneration of sciatic nerve defect in a rat model. The analyses revealed that embedding nanofibrous membranes as topographical cue inside conduits enhanced the recovery rate of the injured nerve more effective than biological and electrical cues as evidenced by functional recovery tests and study of histological changes. These results could provide useful insights into future conduit designs for repairing the injured peripheral nerve. Based on these approaches, in further studies, electrical stimulation should be considered to further improve the nerve repairing procedure.

## Data Availability

The datasets generated during and/or analyzed during the current study are available from the corresponding author on reasonable request.
